# Cultural Competency in Dental Practice: Navigating the Experiences and Perspectives of Dentists in South Punjab, Pakistan

**DOI:** 10.7759/cureus.71322

**Published:** 2024-10-12

**Authors:** Gul Muhammad Shaikh, Saman Baseer, Muhammad Asif Shahzad, Asad Ali, Muhammad Umair Piracha, Amara Nazir, Hafsa Naveed

**Affiliations:** 1 Department of Dental Education and Research, Shahida Islam Medical and Dental College, Lodhran, PAK; 2 Department of Orthodontics and Dentofacial Orthopedics, Sardar Begum Dental Hospital, Gandhara University, Peshawar, PAK; 3 Department of Oral and Maxillofacial Surgery, Azra Naheed Dental College, Superior University, Lahore, PAK; 4 Department of Medical Education, Multan Medical and Dental College, Multan, PAK; 5 Department of Oral Pathology, Nishtar Institute of Dentistry, Multan, PAK; 6 Department of Operative Dentistry, Bakhtawar Amin Medical and Dental College, Multan, PAK; 7 Department of Operative Dentistry and Endodontics, Multan Medical and Dental College, Multan, PAK

**Keywords:** communication barriers, cultural competency, dental practice, dentists, health disparities, pakistan

## Abstract

Introduction

Cultural competence in healthcare embraces the principles of equal access and nondiscriminatory services in healthcare delivery. Dentists as significant healthcare professionals should definitely have the skills needed to diagnose and treat patients’ conditions, but it is crucial to also address nontechnical skills such as cross-cultural communication, the ability to empathize, and inclusive-mindedness. Globally, cultural competency training has been included in healthcare educational programs; however, national practices reportedly have revolved around patients’ clinical competencies and thus entailed overlooking the cultural dimensions of dental education that include diverse sociocultural factors that influence how dental practices are taught, learned, and delivered. These dimensions encompass elements such as language, communication styles, attitudes toward healthcare, and culturally specific oral health beliefs and behaviors. Therefore, the aim of this study is to explore the experiences and perspectives of dentists practicing in South Punjab regarding cultural competency.

Methodology

The study employed a qualitative phenomenological approach. Data was collected through purposive sampling techniques utilizing semistructured interview guides. The participants recruited were licensed dentists practicing in dental institutions in South Punjab, Pakistan. A total of 18 dentists were recruited in the study. The interviews were focused on the different aspects of cultural competency by exploring the perspectives of dentists. Thematic analysis was carried out utilizing the Bran and Clarkes framework to categorize data into key themes and subthemes utilizing inductive coding.

Results

The study revealed that dentists lack awareness and understanding of cultural competency; they also faced significant problems due to limited cultural proficiency while dealing with diverse patients. Major challenges included inadequate training, communication barriers, and cultural bias in dentists while dealing with such diverse populations. Patients recognized the benefits of cultural competency and highlighted facilitators like institution support, peer support, mentorship support, and patient trust. Participants suggested incorporating cultural competency in dentistry curricula, continuous professional development, and community outreach programs for the development of cultural competency among dentists.

Conclusions

The study highlighted perspectives and experiences of dentists regarding cultural competency. The findings demonstrated key problems due to limited or lack of cultural proficiency that included communication and language barriers and unconscious cultural bias among dentists. The study also highlighted facilitators of cultural competency like institutional facilitation, mentoring opportunities from peers and seniors, and patient trust. Facilitating cultural competency among dentists may result in improved patient satisfaction, enhanced patient care, and an increase in compliance, hence overall enhancing the quality of care.

## Introduction

Cultural competency refers to the ability of healthcare professionals to understand, communicate with, and effectively interact with patients from diverse cultural backgrounds. It involves recognizing and respecting cultural differences in values, beliefs, and behaviors that influence patient care, ensuring that services are respectful of and responsive to these differences. Cultural competence in healthcare embraces the principles of equal access and nondiscriminatory services in healthcare delivery. These basic principles should be applied in all circumstances for all patients, regardless of their cultural identities. Cultural identity includes varied characteristics such as those defined by race, ethnicity, national origin, age, gender, socioeconomic status, religion, sexual orientation, disability, religious beliefs, traditions, and even intellectual perspective. All of these characteristics may influence the needs and help-seeking behaviors of individuals and families [1].

Dentists as significant healthcare professionals should definitely have the skills needed to diagnose and treat patients’ conditions, but it is crucial to also address nontechnical skills such as cross-cultural communication, the ability to empathize, and inclusive mindedness. This implies that these qualities of an oral health specialist are important in facilitating rapport and good communication with the patients in as far as enhancing good health is concerned [2]. Organizations like the World Health Organization (WHO) and the Global Dental Council emphasize awareness of health disparities related to race, ethnicity, culture, its interpretation, and policies and strategies to eliminate these disparities in the healthcare system [3]. Similarly, in Pakistan, the Pakistan Medical and Dental Council (PMDC) is responsible for setting the standards that concern medical and dental education. Such standards require competency, skill, and attitudes as well as evidence-based, comprehensive, and patient-centered care, where cultural competence has become an imperative component of the standards [4].

In dentistry, cultural competence is of significant importance because people’s oral hygiene practices, perceptions, and practices differ across cultures. Some beliefs and attitudes toward dental treatment, reporting about pain, and preventive behaviors have cultural variations. Evidence suggests that culturally sensitive dentists are more likely to provide treatment solutions that are acceptable to their patients and therefore are likely to have a higher success rate in terms of the patients’ compliance with treatment regimens. This competency ensures that there is continuity in providing care by filling areas of presumed misunderstandings and providing the dental practitioners with the ability to meet the needs of specific populations [5].

Globally, cultural competency training has been included in the healthcare educational programs in order to equip the professionals for the culturally diversifying patient population. Some of the developed nations, such as the US, Canada, and the UK, incorporated cultural competence topics as part of the dental curricula [6]. These programs aim at fostering and understanding information and skills in healthcare workers to deal with culturally diverse patients. On the other hand, cultural competency has been given some attention in multicultural areas of Pakistan’s healthcare system, but the specific training in this area is scarce, especially in the field of dentistry [7]. National practices reportedly have revolved around patients’ clinical competencies and thus entailed overlooking the cultural dimensions of dental education. Although different studies have explored other competencies of dentists working in different areas of Pakistan, to the best of our knowledge, the area of cultural competency in dentists still remains unexplored in Pakistan and especially in South Punjab [8]. South Punjab is a multicultural region with people of different colors, languages, cultures, and standards of living. Most of these communities have a conventional health system that shapes their perception of dental care. The majority of the population is of rural background, where people use homemade remedies and visit the hospital only when in severe pain [5]. The dentists who are capable of interacting in these cultural terrains can promote trust, preventive care, and reduce the effects of profoundly embedded disparities in health [9].

Therefore, this study aims to address the existing gap by exploring the experiences and perspectives of dentists practicing in South Punjab regarding cultural competency. This research can provide valuable insights for dental educators, policymakers, and practitioners, providing insights and recommendations that can lead to more inclusive and patient-centered care in the region. Ultimately, advocating for culturally competent dental practices fosters a healthcare environment that respects and responds to the diverse needs of all patients.

## Materials and methods

The study employed a qualitative phenomenological approach to explore cultural competency among dentists in South Punjab, Pakistan. A constructivist approach was chosen to examine participants’ understanding, perceptions, and experiences of dentists. The literature search for the development of the semistructured interview guide was conducted using a comprehensive search strategy across databases including PubMed, Scopus, PakMediNet, and Google Scholar search engine. The search incorporated keywords such as "cultural competency," "dental education," "healthcare professionals," "challenges in cultural competency," and "perspectives on cultural competency," combined using Boolean operators like "AND" and "OR" for precision. Studies included in the review were original research articles, published in English between 2014 and 2024. Articles available only as abstracts or citations, conference reports, and editorials were excluded. This approach ensured that the interview guide was developed with content relevance and comprehensiveness for assessing cultural competency in dental professionals. It was followed by internal testing by two members and then expert validation for scrutiny of the appropriateness and comprehensiveness of the content to ensure confirmability and finally field testing for clarity, significance, relevance, and practicality of the questions and to ensure dependability.

Targeted participants included dentists with valid PMDC licenses working in South Punjab. Purposive sampling was used to recruit participants from private and public sector tertiary care dental institutions in South Punjab. Participants were categorized by gender, experience, and professional cadre. Potential participants were contacted by email or WhatsApp and invited to participate. A total of 50 potential participants were invited; however, 28 participants showed willingness to participate, and interviews were conducted with 18 participants after obtaining consent.

The inclusion criteria for this study were as follows: dentists who have completed their house job; practicing in South Punjab; working in PMDC-recognized dental institutions and having a permanent license from PMDC; and those who consented to participate in the study. The exclusion criteria included dentists who are not practicing, those practicing outside South Punjab, those with provisional or no license, and those who did not consent to participate in the study.

Demographic data were collected via an online survey distributed at the time of interview scheduling. Interviews were conducted physically or in person in a private dental clinic. The semistructured interview guide was used by the principal investigator (PI) Gul Muhammad to conduct the interviews, allowing for probing and follow-up questions. Interviews were recorded with participant permission, transcribed verbatim using otter.AI, and anonymized. Transcripts were shared with participants via email or WhatsApp for member checking. All participants accepted the interview contents. Data collection and analysis were carried out simultaneously, and a total of 18 interviews were conducted till data saturation. The length of these interviews ranged from 26 to 35 minutes. One repeat interview was conducted due to technical difficulties in the recording process.

Inductive thematic analysis was used to identify patterns in the data, following the six stages described by Braun and Clarke [10]. This iterative process involved multiple readings of the transcripts, coding, and categorizing the data. Two researchers were responsible for coding the data. Themes and subthemes were derived from the data, and quotations were selected to illustrate and support themes and subthemes. The analysis aimed to describe patterns and identify broader applications of the data.

Ethical approval was obtained from the Institutional Review Board/Independent Ethical Committee of Multan Medical and Dental College with approval number C-78-1032. Participants were provided with detailed information about the study's purpose, procedures, and rights, including the right to withdraw at any time without consequences. Informed consent was obtained from all participants prior to data collection. Confidentiality and anonymity were maintained by assigning pseudonyms to participants and securely storing all data accessible only to the research team. Consolidated criteria for reporting qualitative research (COREQ) were used to guide the work and improve the reporting of findings [11].

## Results

The study included 18 dentists from dental institutions in South Punjab. Table 1 provides a summary of the demographic characteristics of the participants. Of the 18 participants, 10 were male (55.6%) and eight were female (44.4%). Participants’ ages were distributed as follows: 25-34 years (six participants, 33.3%), 35-44 years (eight participants, 44.4%), and >45 years (four participants, 22.2%). The academic positions were categorized into professors (two participants, 11.1%), associate professors (four participants, 22.2%), assistant professors (five participants, 27.8%), postgraduate residents (two participants, 11.1%), and demonstrators (five participants, 27.8%). Regarding years of teaching experience, four participants had 2-5 years (22.2%), five had 6-10 years (27.8%), six had 11-15 years (33.3%), and three had more than 15 years (16.7%). The participants were from both public (six participants, 33.3%) and private (12 participants, 66.7%) institutions.

**Table 1:** Demographic characteristics of the participants

Characteristics	Frequency (n)	Percentage (%)
Gender		
Male	10	56
Female	8	44
Age range (years)		
25-34	6	33
35-44	8	45
>45	4	22
Academic position		
Professor	2	11
Associate professor	4	22
Assistant professor	5	28
Postgraduate resident	2	11
Demonstrator	5	28
Experience (years)		
2-5	04	22
6-10	05	28
11-15	06	33
>15	03	17
Institution type		
Public	06	33
Private	12	67

The qualitative analysis of this study revealed four major themes related to cultural competency in dental practice: understanding cultural competency, challenges in cultural competency, facilitators of cultural competency, and recommendations for improvement. These themes are supported by subthemes that explore the perspectives and experiences of dentists practicing in South Punjab. The findings highlighted the critical role of cultural competency in enhancing patient care and the need for structured interventions to improve these skills among dental practitioners.

Findings highlighted a relatively diverse level of awareness of cultural proficiency and its implications on dental practice. While participants recognized the significance of awareness and proficiency in cultural sensitivity. Respondents also acknowledged that they experienced patients from different cultural backgrounds and were unaware of their cultural beliefs regarding specific issues and treatment options due to a lack of training on cultural competency. Moreover, most respondents stated that there was no cultural competency content during their college years as the curriculum was focused mainly on technical competence with little attention to cultural aspects. Participants also pointed out that differences in culture and language act as a barrier to good patient relations and might affect compliance.

Most of the participants highlighted a lack of structured cultural competency training programs and opportunities for undergraduate students and faculty as a key barrier for cultural proficiency. Respondents also believed that communication barriers were due to a lack of proficiency in local languages that affected communication of technical aspects of certain procedures to these patients. Moreover, participants highlighted potential unconscious bias and stereotypical behavior for patients that affected the outcomes.

Participants also highlighted the factors that facilitated cultural competency. Respondents highlighted that the institutional support and programs like workshops were helpful in enhancing the competency of cultural proficiency. Support from peers and mentors was also identified as an important factor in increasing cultural sensitivity; several participants pointed out that their supervisors taught them how to act in diverse cultural situations. Furthermore, respondents highlighted that understanding patients cultural beliefs and sensitivity helped in building trust and ultimately good compliance.

The findings identified strategies to enhance the cultural competency of dentists. Respondents highlighted the need for the integration of cultural competence modules in the undergraduate curriculum so that there is a good preparation for handling patients from diverse cultural backgrounds. Moreover, participants also suggested incorporating cultural competency training as part of continuous professional development (CPD) programs. Furthermore, participants also pointed to the need for frequent community outreach programs to enhance their understanding of different cultures and languages (Table 2).

**Table 2:** Dentists perspectives and experiences regarding cultural competency M: Male; F: female; G: government; P: private; R: respondent; 1-18: respondent index; 25-55: age range

Themes	Subthemes	Representative quotations
Understanding cultural competency	Awareness of cultural diversity	"I realized that patients specially from rural backgrounds have specific cultural beliefs about dental health that I wasn’t aware of." (M34PR07)
	Cultural training in dental education	"During my dental college years, cultural sensitivity was never a priority; we only learned about clinical aspects." (F30PR013)
	Impact of cultural differences on patient care	"Sometimes, lack of awareness of cultural beliefs and language barriers make it hard to explain treatment plans to patients, affecting trust and compliance." (M38GR09)
Challenges in cultural competency	Limited cultural competency training	"There is no formal training on how to handle diverse and marginalized patient populations, and we mostly rely on personal experiences." (M41PR16)
	Communication barriers	"Communicating with patients who don't speak the same language(urdu or English) was very difficult, especially when explaining technical procedures." (F47GR03)
	Stereotyping and bias	"In some cases, dentists themselves hold certain biases that they’re unaware of, which affects their patient interactions." (M35PR14)
Facilitators of cultural competency	Institutional support for cultural sensitivity	"Our dental college organized few workshops on social accountability in which we learnt about cultural competency and that was very helpful.” (M28GR17)
	Mentorship and peer support	"As I was not the day scholar senior dentists and colleagues often helped me in dealing complex cultural issues with patients." (F48PR15)
	Patient-provider trust	"When patients see that I understand their cultural background, it immediately builds trust and makes the consultation smoother." (M37GR08)
Recommendations for Improvement	Inclusion of cultural competency in curriculum	"There needs to be a dedicated module on cultural competency in our dental education to prepare future practitioners." (M28PR10)
	Continuous professional development (CPD) on cultural sensitivity	"Workshops on cultural competency should be part of CPD so we can continually improve our skills." (F41PR12)
	Community engagement	"Dentists should be encouraged to engage with the communities they serve to understand their unique cultural needs better." (M33GR11)

## Discussion

The study explored cultural competency in dental practice through the experiences and perspectives of dentists in South Punjab Pakistan. The findings provided insights into the current level of understanding, barriers, facilitators, and areas for improvement for enhancing cultural proficiency in dentists.

The study revealed that dentists are not completely aware of and prepared themselves in terms of cultural proficiency. The findings are supported by a previous study conducted in Iraq in 2024 that also pointed out a limited understanding of cultural competency by health professionals [12]. The study also showed a major deficiency in formal cultural competence training for dentists at both undergraduate and postgraduate levels, consistent with previous studies pointing out the marginal concentration on cultural proficiency within the healthcare curricula. According to a study from Australia, cultural competency lacks priority in dental curricula, making practitioners insufficiently equipped to deal with diverse patient populations [13]. The findings suggest organized incorporation of cultural competency in dental education.

Language as a form of communication barrier was mentioned by most of the participants as one of the biggest challenges they face in their practice due to limited cultural training. This is consistent with another study conducted in the UK in 2018 that established that language barriers are likely to affect effective patient interactions [14]. This issue was particularly prominent in the resource-constrained setting of South Punjab, Pakistan, where limited access to financial, educational, and infrastructural resources complicates the effective implementation of cultural competency training. Furthermore, the diverse linguistic landscape, with varying languages and dialects across regions, poses additional challenges in patient communication and care, necessitating greater institutional support and targeted training programs to overcome these barriers. The participants highlighted that they faced problems explaining procedures to the patients who spoke different local languages, as this hindered the patient’s understanding and confidence with the dentist. The findings suggest that language training should be a part of cultural competency training. The research also highlighted that dentists may have unconscious cultural bias but are not able to manage it due to a lack of training. In a similar study on implicit bias in healthcare, it was found that while clinicians do recognize biases when present in themselves, they also fail to know how to mitigate them in practice [15]. 

The study also highlighted the need for institutional support in developing cultural competency among dentists. These challenges are in line with a previous study conducted in 2019 that highlighted dental and oral health students recognize the need for increased institutional support to develop cultural competence [16]. The findings of the study suggest targeted professional development plans for dentists at the institutional level to cater to these issues. Participants in the study emphasized the importance of mentorship and peer support in developing cultural competency. They highlighted the support they received from peers and mentors while handling issues of cultural sensitivity. The findings are endorsed by a study conducted in 2017 that stated peers and seniors play a vital role in enhancing the cultural competency of health professionals [17].

The study findings also suggested future improvements to incorporate the cultural competence courses in programs of dentistry. The participants also expressed the need to promote CPD in cultural competency areas to facilitate the development of these skills. The participants also exhibited significant interest in the development of cultural competency to understand and treat their patients well. This is in concurrence with a past study that has urged that cultural competency be seen as an evolving developmental continuum rather than a one-time training category [18].

Moreover, the study also concluded that increased community outreach programs could be a productive approach to cultural sensitivity. Participants highlighted the significance of working closely with patients in the local communities for developing communicational and cultural sensitivity skills. The results are validated by a previous study conducted in Spain in 2019 that showed that exposure to community context can greatly enhance cultural competence among health personnel [19].

This study presents several notable strengths that significantly enhance the understanding of cultural competency within dental education in Pakistan. Employing a qualitative phenomenological approach allowed for a nuanced exploration of the perspectives and experiences of dentists practicing in South Punjab, yielding rich, contextually relevant data that illuminate the multifaceted nature of cultural competency in dental practice. The diverse participant pool, including general dentists, specialists, and postgraduate trainees from both public and private institutions, enhances the study's representativeness and applicability, offering a comprehensive view of the challenges and facilitators associated with cultural competency across different contexts. Furthermore, the study addresses a critical gap in the literature by focusing on South Punjab, providing valuable insights into the sociocultural dynamics that impact dental education and practice in this unique geographical setting. The identification of specific barriers and facilitators, such as institutional support and mentorship, underscores the importance of context-specific strategies for enhancing cultural competency in dental education. 

Limitations

For an in-depth understanding of dentists perspectives, a qualitative design was utilized, and a small sample size may limit the generalizable value of the findings. Moreover, the self-reported data provided by the participants may have resulted in response bias, and the respondents may have provided responses that were deemed socially acceptable. Besides, the study focused on the South Punjab region only; hence, it might not represent the experiences and perspectives of the dentists of other regions of Pakistan or any part of the world. Furthermore, although the qualitative design of the study provided in-depth exploration of the topic, it is difficult to quantify the findings. Future studies should include a more diverse population; emphasizing a mixed-methods approach could validate and build upon these findings. The involvement of students and administration might help to provide a broader picture of cultural competency among dentists.

## Conclusions

The study highlighted the perspectives and experiences regarding cultural competency among practicing dentists in Pakistan. The research revealed that dentists have a low level of understanding and awareness of cultural competency. Additionally, there was a lack of training opportunities at the institutional level that affected the trust and compliance of patients. The findings demonstrated key problems resulting from ineffective structured cultural competency among dentists that included communication problems due to limited understanding of local languages and implicit cultural biases that remain unaddressed. The study also emphasized the factors that enhanced cultural competency among dentists, including support from institutions, opportunities for mentorship, and the establishment of trust with patients. The study also highlighted areas for improvement, such as the inclusion of a cultural competency framework in the dental curriculum, providing targeted training for faculty, and fostering community engagement. By implementing these changes, the institutions can foster cultural competency and contribute to the quality and effectiveness of the healthcare system.

## Appendices

**Table 3:** Semistructured interview guide for dentists

Question number	Interview questions
1	Describe your understanding of cultural competency in dental practice. (a) How do you think it's linked for patient care, especially in Pakistan?
2	How often do you encounter patients from diverse cultural backgrounds? Can you share examples where cultural differences impacted dental care?
3	How well-prepared do you feel in addressing the cultural needs of your patients? Have you received any formal training on cultural competency?
4	What challenges have you faced in practicing culturally competent care, particularly in underserved settings?
5	What role do your institution's policies and resources play in facilitating cultural competency in your practice?
6	How do you adapt your communication strategies when dealing with patients from different cultural or linguistic backgrounds?
7	To what extent are you or your institution involved in community outreach or engagement to address cultural competency?
8	What could be done to improve cultural competency training for dentists in rural areas? What specific steps would you recommend?
9	How do you think cultural competency affects patient satisfaction, treatment outcomes, and overall dental care delivery in rural areas?
10	What changes or initiatives would you suggest to better integrate cultural competency into dental practice in underserved or rural regions?
